# Genetic Diversity and Population Structure of *Acanthopagrus latus* in the South China Sea

**DOI:** 10.3390/ani15091295

**Published:** 2025-04-30

**Authors:** Cheng-He Sun, Qun Zhang, Chang-Hu Lu

**Affiliations:** 1 College of Life Sciences, Nanjing Forestry University, Nanjing 210037, China; luchanghu@njfu.com.cn; 2 Department of Ecology and Institute of Hydrobiology, Jinan University, Guangzhou 510632, China

**Keywords:** phylogeny, population structure, South China Sea, stock enhancement, wild populations, yellowfin seabream (*Acanthopagrus latus*)

## Abstract

The yellowfin seabream *Acanthopagrus latus* (Houttuyn, 1782) belongs to the order Spariformes and family Sparidae and is tender, rich in fat, and nutritious, making it a high-value seafood variety along the China coast. The fry can be used for large-scale aquaculture in both seawater and freshwater. Due to environmental pollution and excessive fishing, natural *A. latus* populations have declined significantly in recent years, which severely depletes genetic diversity. Here, we analyzed the genetic structure of four wild *A. latus* populations in the South China Sea to provide insights into the genetic resources. In particular, we analyzed 40 samples from four sampling sites through whole-genome resequencing, yielding 515 Gb of raw data and 132,505,081 single nucleotide polymorphisms. Population structure, phylogenetic, and principal component analyses revealed that the four populations were homogeneous and did not show clustering based on geographical origin, indicating extensive admixture. This could be attributed to recent enhancement and release activities in the South China Sea. This information may help to protect and utilize *A. latus* germplasm resources.

## Simple Summary

The Yellowfin seabream *Acanthopagrus latus* (Houttuyn, 1782) belongs to the order Spariformes and family Sparidae and is widely distributed in the Indo-Northwest Pacific, extending from the Persian Gulf in the west to the Philippines in the east, to Japan in the north, and to Australia in the south. Here, we analyzed the genetic structure of four wild *A. latus* populations in the South China Sea to provide insights into their genetic resources. Four populations were analyzed using whole-genome resequencing. Principal component analysis, phylogenetic tree construction, and population structure analysis revealed that individuals from different geographical populations were mixed and not clustered according to geographical location, indicating extensive gene exchange between populations; however, this does not rule out the impact of stock enhancement in the South China Sea in recent years.

## 1. Introduction

The Yellowfin seabream *Acanthopagrus latus* (Houttuyn, 1782) is widely distributed in the Indo-Northwest Pacific, extending from the Persian Gulf in the west to the Philippines in the east, Japan in the north, and Australia in the south [1,2]. Classified in the perciform family Sparidae, *A. latus* is a small- to medium-sized fish found in shallow, warm-temperate waters, with a typical body length of 200–300 mm [3,4,5]. It has a wide salt tolerance range and can survive in seawater with a salinity of 0.5~4.3‰. Occasionally, it enters estuaries or freshwater areas, and young fish mostly inhabit gentle, semi-saline waters within the bay [5,6,7]. It is omnivorous and benthic carnivorous, feeding mainly on polychaetes, mollusks, crustaceans, echinoderms, and other small fish species. This species does not present long-distance migratory behavior but exhibits noticeable reproductive migration. Approximately 2 months before the spawning period, it begins to move from nearshore brackish water to high-salinity deep-sea regions and returns to nearshore areas after spawning. The reproductive period is from October to January, and the peak spawning period is from November to December. From January to February of the following year, many juvenile fish appear at the intersection of ports and brackish water, and many natural fry can be caught in this period [1,2,5].

The flesh of *A. latus* is rich in fat and has high nutritional value. Therefore, it is considered a high-value seafood variety along the coast of China [6,7]. After domestication, the fry can be used for large-scale aquaculture in both seawater and freshwater. In the 1980s, advances in artificial breeding techniques for Chinese *A. latus* promoted the development of the saltwater and brackish freshwater aquaculture of this species. In some countries and regions with developed aquaculture industries, *A. latus* has become the main aquaculture species [6,7]. However, its breeding cycle is relatively long, and it takes 1.5–2 years to reach the market specification of approximately 250 g. In recent years, owing to environmental pollution and excessive fishing, natural *A. latus* populations have declined significantly, resulting in the severe depletion of genetic diversity [6,7]. The breeding population of *A. latus* also faces serious challenges associated with breeding germplasms owing to inbreeding and small-scale parental artificial breeding. Genetic diversity in natural populations can be influenced by the proliferation and release of aquatic organisms [8]; for example, the release of Chinese shrimp fry from the Shandong Peninsula has a complementary effect on their wild resources [9]. Regarding actual breeding and release activities, unmanaged stocking could have adverse effects on the genetic structure, species diversity, and ecosystem structure.

Analysis of long-term changes in the diversity and structure of *A. latus* populations is necessary to restore and protect resources. Population genetic analyses of *A. latus* are limited to analyses of the mitochondrial control region. Liu et al. [10] evaluated genetic polymorphism in the mitochondrial D-loop gene of three populations of *A. latus* in Xiamen, Zhuhai, and Haikou. Xia et al. [11] analyzed genetic diversity in a population of *A. latus* along the coast of South China based on the D-loop region, showing that the population could be divided into two groups with the Qiongzhou Strait as the boundary.

Whole-genome resequencing is an efficient method for obtaining genetic information from samples [12,13,14]. The main goal is to evaluate individuals (usually from different populations or regions) of a species with published reference genome sequences based on whole-genome sequencing [15]. This approach can provide insight into interspecific differences at the whole-genome sequence level and reveal the role of genetic variation in biological processes, molecular structures, and cellular components [12,16]. Population resequencing typically involves the whole-genome resequencing of multiple individuals for population-level analyses [17]. The accuracy and precision of genetic variation detected using population resequencing are higher than those using individual resequencing [18]. Therefore, population resequencing is an important tool in genomics and is frequently used to study changes in gene and genotype frequencies within a population, providing a basis for resolving various issues, such as environmental adaptability [19].

In this study, whole-genome resequencing data from four populations are used to evaluate the genetic structure of *A. latus* using various approaches, including principal component, population structure, and phylogenetic analyses. The aim of this study is to provide scientific data for the development, utilization, and conservation of *A. latus* genetic resources.

## 2. Materials and Methods

### 2.1. Sample Collection and DNA Extraction

**Figure 1:**
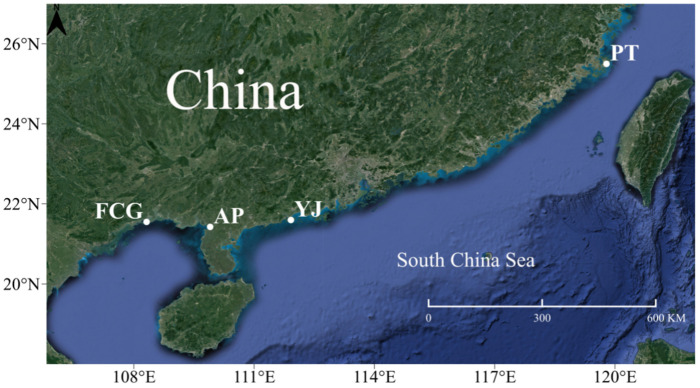
Sampling sites for *Acanthopagrus latus*.

In this study, a total of 40 samples (ten samples from four different wild *A. latus* populations, with samples collected randomly) were purchased from local fishers with commercial fishing permits. Samples were collected from Pingtan, Fujian (PT), Yangjiang, Guangdong (YJ), Anpu, Guangxi (AP), and Fangchenggang, Guangxi (FCG) (Table 1; Figure 1). Fresh samples were identified based on morphological features and numbered according to the geographical location. The back muscles of the fish were then dissected, cut, and immersed in anhydrous ethanol at −20 °C for storage. All specimens in this study were collected in accordance with Chinese laws. Specimen collection was reviewed and approved by the Animal Ethics Committee of Jinan University (No. jnu20230109.2). The phenol–chloroform method [20] was used for genomic DNA extraction, and the extracted DNA samples were stored at −80 °C. The concentration and mass of the total genomic DNA were determined using a NanoDrop 2000 spectrophotometer (Thermo Fisher Scientific, Waltham, MA, USA).

### 2.2. Whole-Genome Resequencing

Sequencing was performed by Wuhan BENAGEN Technology Co., Ltd. (Wuhan, China) Filtering and quality control were also completed by Wuhan BENAGEN Technology Co., Ltd., (Wuhan, China) and performed in accordance with the company’s standardized procedures. High-quality, minimally degraded DNA with good continuity was randomly sheared. Using the standard protocol provided by the second-generation sequencing company, a DNA library (350 bp) was constructed for Illumina sequencing for each sample [21]. After DNA library construction, double-terminal sequencing was performed on the Illumina HiSeq 2000 platform (Illumina, San Diego, CA, USA) with a read length of 150 bp. Raw reads were filtered to obtain high-quality sequences for subsequent analyses.

### 2.3. Data Analysis

Genome sequencing data after quality control were compared with the reference genome for *A. latus* (RefSeq: GCF_904848185.1, https://www.ncbi.nlm.nih.gov/datasets/genome/GCF_904848185.1/, accessed on 23 December 2023) using BWA-MEM2 v2.2 [22], with default parameters. After quality control, the BWA-MEM algorithm was used to compare sequencing reads with the reference genome of *A. latus*. The mapping results were sorted, and duplicate reads were removed using samtools v1.9. The HaplotypeCaller, CombineGVCFs, GenotypeGVCFs, and SelectVariables modules in GATK v4.2.0 were used to obtain single nucleotide polymorphism (SNP) and insertion/deletion information, with standard parameters. Subsequently, the mutation information was filtered using the VariantFiltration module. Annovar and SnpEff were used to annotate the SNPs [23,24].

MEGA v5.0 [25] was used to create alignments. Based on the neighbor-joining method, a p-distance–based phylogenetic tree was constructed. Using EIGENSOFT v5.01 [26], principal component analysis (PCA) was conducted based on SNP data to evaluate the clustering of samples. The population structure was analyzed using ADMIXTURE v1.3.0 [27], setting the number of subgroups (K-value) to 1–10 for clustering and using cross-validation to determine the optimal number of subgroups based on the valley value of the cross-validation error rate. Pairwise genetic relationships between individuals were estimated using GCTA [28], and linkage disequilibrium was analyzed using Plink v1.07 [29]. Using VCFtools v0.1.16 [30], we calculated various population genetic indicators (Ti/Tv, heterozygosity, and homozygosity) according to the specified window (100 kb) and step size (10 kb).

## 3. Results

### 3.1. Sequencing Data Statistics

Using the Illumina HiSeq 2000 platform, a double-terminal sequencing library was constructed and sequenced based on 40 *A. latus* specimens. In total, 515 Gb of raw sequencing data were obtained. After quality control (Table 2), the clean reads accounted for 99.11% of all sample data. The average Q30 value (i.e., the proportion of bases with a Phred mass value greater than 30 relative to the total bases) was 93.61%. Therefore, the resequencing data in this study exhibited good quality and high accuracy and could be used for further analyses.

For the 40 samples, the average proportion of reads that matched the reference sequence was 97.21%, the average sequencing depth was 14.13×, and the average sequencing coverage was 99.42%. Specific information regarding resequencing data mapping is presented in Table 3.

### 3.2. SNP Detection

In total, 132,505,081 SNPs were detected in the 40 samples. The number of transitions ranged from 1,712,297 to 6,368,531, and the number of transversions ranged from 982,734 to 3,994,927. The transition-to-transversion ratio was 1.59–1.74. The number of heterozygous mutations was 1,925,968–6,760,257, and the number of homozygous mutations was 734,866–3,603,201 (Table 4).

### 3.3. Phylogenetic Analysis and PCA

**Figure 2:**
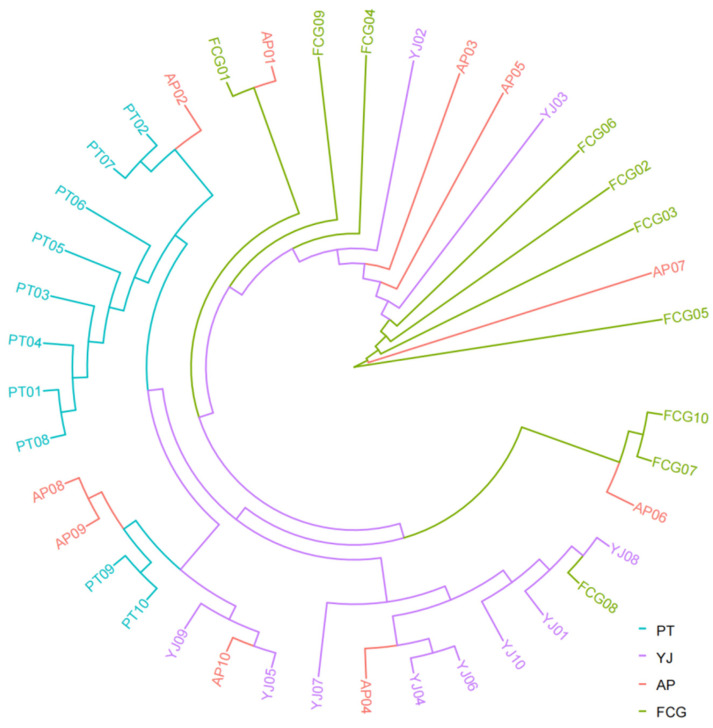
Neighbor-joining phylogenetic tree based on whole-genome single-nucleotide polymorphism (SNP) data for *Acanthopagrus latus* populations.

**Figure 3:**
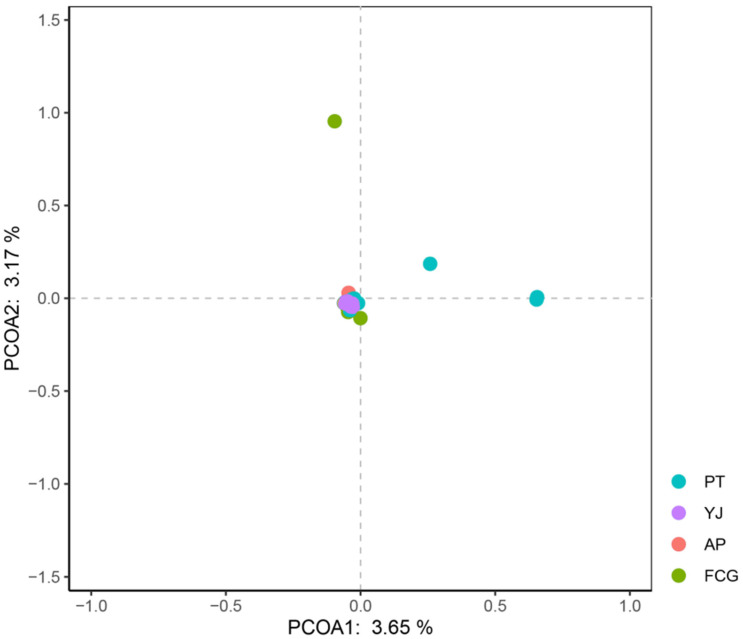
Principal component analysis (PCA) plot of *Acanthopagrus latus* samples.

All samples of *A. latus* clustered together (Figure 2), irrespective of geographical origin (indicated by different colors). Although most individuals in the PT population clustered in a single branch, individuals in the AP and YJ populations were interspersed. PCA of the *A. latus* samples indicated that most individuals in the four populations clustered together, while the explanatory power of the first two principal components was very low. This was consistent with the results of the phylogenetic analysis (Figure 3). Both the FCG and PT populations included individuals that did not cluster well with other individuals.

### 3.4. Population Structure and Linkage Disequilibrium Analysis

**Figure 4:**
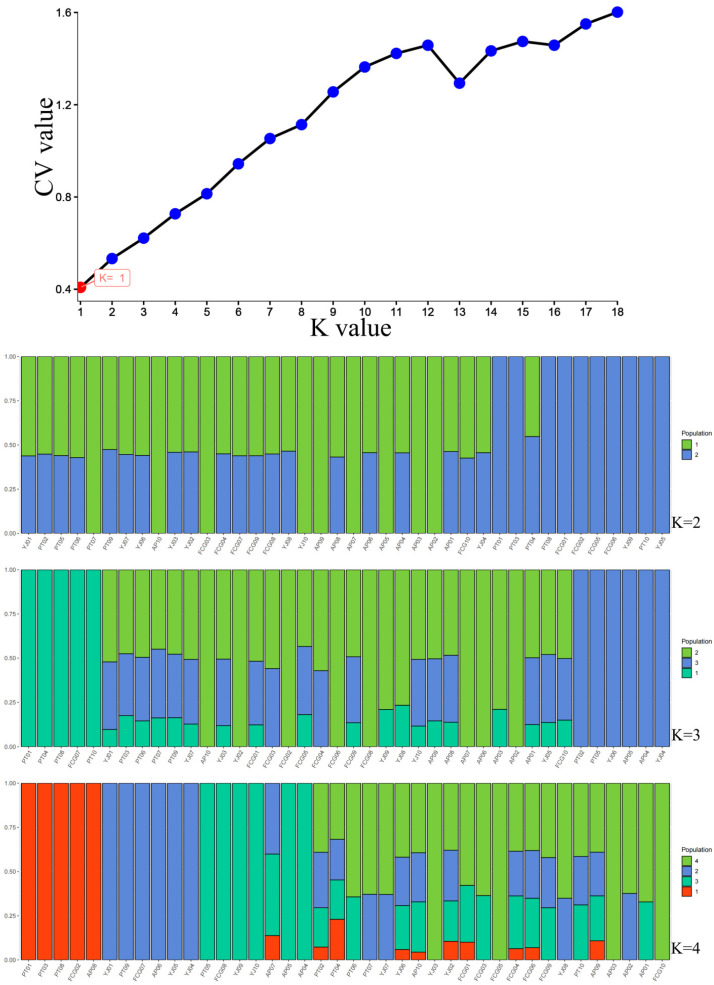
Population structure plots with K = 2 to 4 for *Acanthopagrus latus* populations.

**Figure 5:**
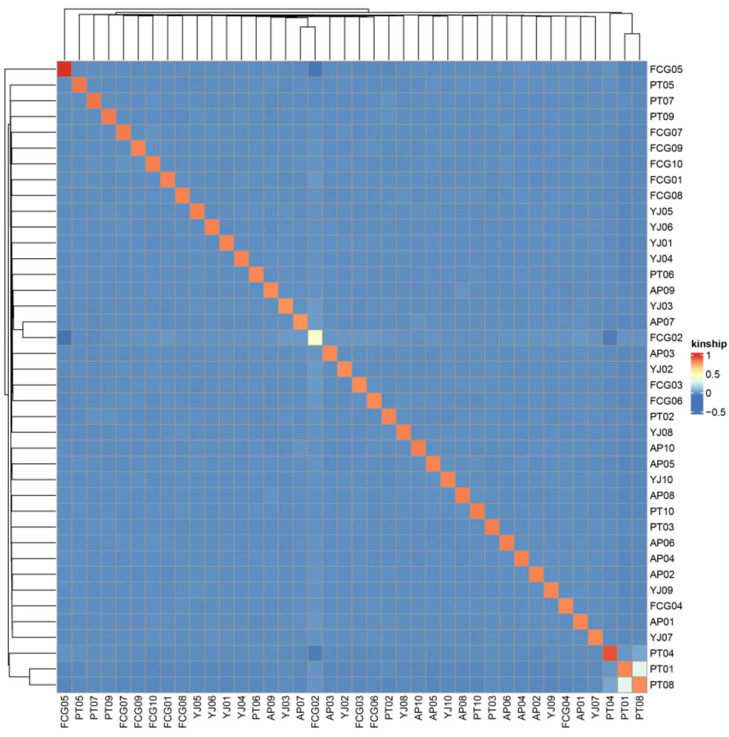
Visualization of G-matrix analysis results for *Acanthopagrus latus* populations.

**Figure 6:**
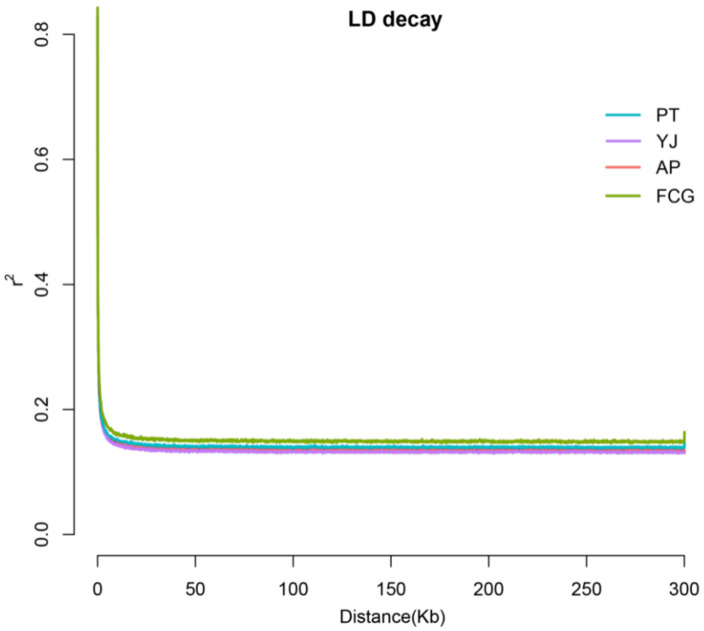
Linkage disequilibrium decay for *Acanthopagrus latus* populations.

Cross-validation error rates for the number of clusters (K) between one and 18 exhibited an upward trend (Figure 4). The cross-validation error rate was the smallest when K = 2. The optimal number of clusters was K = 1, indicating that the samples shared one gene library. The population structure analysis results (Figure 4) were consistent with the phylogenetic tree and PCA results. All individuals belonged to a single, large South China Sea population and showed a high degree of kinship among individuals (Figure 5). The linkage disequilibrium (LD) (Figure 6), based on pairwise r^2^ values, indicated that LD coefficients for the populations decreased in the following order: FCG > PT > AP > YJ. The decay rates from fast to slow were as follows: YJ > AP > PT > FCG.

## 4. Discussion

In this study, our goals included determining the genetic diversity and population structure of A. latus in the South China Sea. The average sequencing depth and coverage were high, indicating that our sequencing data could accurately reflect genetic variation in *A. latus*. The transition-to-transversion ratio for SNPs was >1.5, indicating that *A. latus* exhibits transformational reversal bias, as observed in most vertebrates [31]. Admixture analysis suggested that the four populations were “completely admixed”. Clustering analyses, PCA, and phylogenetic analyses suggested that the four populations could be collectively referred to as the South China Sea population.

Genetic diversity is the foundation for species survival, adaptation, and evolution [32,33]. High genetic diversity within a species leads to an enhanced ability to adapt to the environment and improves evolutionary potential [34]. Extensive enhancement and release activities have been conducted in the South China Sea in recent years [3,4,5]. The fact that all four populations were from the South China Sea may have resulted from these activities.

He et al. [35] conducted a comprehensive genetic diversity analysis of eight populations of *A. latus* along the coast of South China, using mitochondrial control region sequences. They detected high overall genetic diversity and identified the populations east and west of the Qiongzhou Strait as two management units, consistent with previous results. Many provinces and cities in China have attempted to breed and release *A. latus*; however, recent reports on the genetic diversity of natural populations of *A. latus* are limited [35]. Genetic information on changes in the wild germplasm resources of this species after release is also lacking, hindering management and conservation.

Many artificially bred fry have entered natural waters. If the genetic diversity of the released population is significantly lower than that of the wild population, large-scale breeding and release activities are likely to reduce the overall genetic diversity of the species, thereby affecting its sustainable development. This can occur if the released population is derived from a small number of parents or if the contribution rate of gametes from different parents to offspring is imbalanced [36], in which case the genetic diversity of the released population will generally be lower than that of the wild population. In addition, fitness is highly correlated with genetic diversity, and the fitness of a released population is influenced by the genetic diversity of its parents [36]. If fry with low fitness are introduced in natural waters, hybridization between the released and wild populations may increase the frequency and expression of harmful recessive genes in the wild population, ultimately leading to species degradation. A decrease in the genetic diversity due to stocking can lead to an increase in harmful recessive gene expression, a decline in certain adaptive traits, and consequent changes in population fitness, resulting in problems such as low survival rates, weak reproductive ability, slow growth, and poor adaptability [37,38]. Therefore, the diversity of parental strains for fry breeding should not be lower than that of wild populations, if possible, and the number of parents subjected to breeding should be maximized to avoid reductions in genetic diversity and fitness. Blankenship and Leber [39] suggested the implementation of genetic monitoring and management of parents and released populations when evaluating the effectiveness of proliferation and release.

## 5. Conclusions

Overall, there were no significant differences among the four populations of *A. latus*, indicating significant homogenization. Various techniques (e.g., phylogenetic analysis, clustering, PCA, and kinship analysis) showed that the four populations formed a well-supported cluster. These results suggest that proliferation and release activities have played a crucial role in shaping the genetic structure of *A. latus* populations. This study provides an important reference for the protection and utilization of germplasm resources of this species.

## Figures and Tables

**Table 1:** Information on *Acanthopagrus latus* sample collection.

Sampling Site	Sample No.	Coordinates	Sample Size	Sample Date
Pingtan, Fujian	FT01-10	119.80° E, 25.52° N	10	4/2021
Yangjiang, Guangdong	YJ01-10	111.84° E, 21.58° N	10	12/2021
Anpu, Guangdong	AP01-10	109.92° E, 21.43° N	10	12/2021
Fangchenggang, Guangxi	FCG01-10	108.36° E, 21.76° N	10	4/2021

**Table 2:** Quality control for resequencing data.

Samples	Total Reads	Clean Reads	Percentage of Clean Reads	Clean Bases	GC Content	%>Q20	%>Q30
PT01	101,809,808	100,964,806	99.17%	15,071,611,232	41.84%	97.72%	93.39%
PT02	76,468,810	75,751,664	99.06%	11,308,412,169	41.78%	97.71%	93.47%
PT03	87,559,762	86,760,620	99.09%	12,960,643,583	41.77%	97.53%	93.01%
PT04	68,839,290	68,220,428	99.10%	10,177,228,391	41.64%	97.52%	92.92%
PT05	75,535,398	74,971,184	99.25%	11,191,679,420	41.83%	97.82%	93.61%
PT06	67,075,948	66,449,744	99.07%	9,913,777,075	41.87%	97.45%	92.81%
PT07	108,258,312	107,473,016	99.27%	16,047,612,530	41.77%	98.04%	94.15%
PT08	89,303,552	88,674,560	99.30%	13,244,598,365	41.60%	98.01%	94.07%
PT09	86,840,376	86,052,666	99.09%	12,836,784,979	41.91%	97.63%	93.24%
PT10	89,958,686	89,252,390	99.21%	13,320,160,715	41.64%	97.79%	93.55%
YJ01	90,160,316	89,401,880	99.16%	13,330,989,547	41.95%	98.00%	94.09%
YJ02	57,567,502	57,051,926	99.10%	8,517,653,507	41.91%	97.50%	92.94%
YJ03	47,270,776	46,781,760	98.97%	6,981,618,750	42.07%	97.53%	93.07%
YJ04	88,343,852	87,589,348	99.15%	13,066,629,055	41.87%	97.68%	93.39%
YJ05	82,130,622	81,490,468	99.22%	12,152,811,875	42.07%	98.04%	94.21%
YJ06	89,258,334	88,489,374	99.14%	13,207,771,049	41.96%	97.54%	92.96%
YJ07	137,332,282	135,238,024	98.48%	18,602,555,953	42.16%	97.41%	93.33%
YJ08	184,719,214	183,261,698	99.21%	27,348,916,620	41.88%	98.07%	94.25%
YJ09	69,634,448	68,875,348	98.91%	10,281,000,273	42.27%	97.47%	92.88%
YJ10	191,434,786	189,944,896	99.22%	28,352,038,411	41.36%	98.05%	94.20%
AP01	61,502,906	60,875,958	98.98%	9,090,671,085	41.90%	97.62%	93.26%
AP02	70,704,218	70,013,212	99.02%	10,460,754,702	41.88%	97.37%	92.66%
AP03	52,874,558	52,325,704	98.96%	7,819,456,120	42.07%	97.48%	92.92%
AP04	81,233,154	80,524,948	99.13%	12,031,774,277	41.82%	97.75%	93.51%
AP05	76,994,054	76,440,744	99.28%	11,290,946,422	39.96%	97.91%	93.87%
AP06	69,974,798	69,337,560	99.09%	10,362,493,909	42.12%	97.62%	93.15%
AP07	41,426,420	40,999,866	98.97%	6,122,846,167	42.14%	97.36%	92.63%
AP08	86,534,524	85,750,702	99.09%	12,808,516,569	42.26%	97.97%	94.00%
AP09	79,513,196	78,778,208	99.08%	11,769,256,492	42.23%	97.86%	93.75%
AP10	81,170,812	80,450,254	99.11%	12,024,905,820	42.29%	97.93%	93.91%
FCG01	84,932,016	84,282,552	99.24%	12,537,445,295	41.93%	97.99%	94.03%
FCG02	81,907,400	81,263,586	99.21%	12,092,232,810	41.76%	97.86%	93.62%
FCG03	61,745,044	61,744,694	100.00%	9,125,633,721	38.21%	97.62%	93.05%
FCG04	83,019,154	82,443,344	99.31%	12,167,842,428	41.07%	98.05%	94.21%
FCG05	114,709,826	112,695,646	98.24%	16,821,503,661	41.98%	98.10%	94.55%
FCG06	82,966,560	82,035,818	98.88%	12,212,423,001	41.70%	97.73%	93.56%
FCG07	103,097,888	102,132,854	99.06%	15,113,755,765	41.89%	98.18%	94.82%
FCG08	120,985,092	119,862,310	99.07%	17,422,493,980	41.79%	98.13%	94.77%
FCG09	86,941,476	86,193,752	99.14%	12,771,778,691	41.05%	98.16%	94.66%
FCG10	90,569,546	89,881,584	99.24%	13,344,103,638	41.26%	97.92%	94.10%

**Table 3:** Results of re-sequencing data mapping.

Sample	Total Reads	Mapped	Mapping Rate (%)	Depth (X)	Coverage (%)
PT01	100,964,806	99,759,774	98.81	17.42	99.59%
PT02	75,751,664	74,968,419	98.97	13.82	99.48%
PT03	86,760,620	85,869,957	98.97	15.35	99.55%
PT04	68,220,428	67,098,513	98.36	11.35	99.54%
PT05	74,971,184	73,679,321	98.28	12.57	99.48%
PT06	66,449,744	65,678,985	98.84	12.16	99.47%
PT07	107,473,016	105,097,491	97.79	17.1	99.61%
PT08	88,674,560	87,780,571	98.99	14.87	99.53%
PT09	86,052,666	85,194,994	99.00	14.88	99.54%
PT10	89,252,390	88,455,359	99.11	15.52	99.54%
YJ01	89,401,880	88,135,270	98.58	14.98	99.49%
YJ02	57,051,926	56,647,410	99.29	10.77	99.37%
YJ03	46,781,760	46,405,601	99.20	8.69	99.29%
YJ04	87,589,348	86,906,921	99.22	15.33	99.50%
YJ05	81,490,468	80,833,049	99.19	14.01	99.45%
YJ06	88,489,374	87,826,158	99.25	15.24	99.52%
YJ07	135,238,024	134,656,295	99.57	19.5	99.54%
YJ08	183,261,698	180,627,066	98.56	30.77	99.60%
YJ09	68,875,348	68,316,440	99.19	12.16	99.47%
YJ10	189,944,896	185,373,476	97.59	31.47	99.63%
AP01	60,875,958	60,412,288	99.24	11.11	99.42%
AP02	70,013,212	69,428,852	99.17	12.83	99.44%
AP03	52,325,704	51,934,968	99.25	9.81	99.31%
AP04	80,524,948	79,950,872	99.29	14.22	99.47%
AP05	76,440,744	74,675,889	97.69	12.81	99.32%
AP06	69,337,560	68,755,802	99.16	11.75	99.41%
AP07	40,999,866	40,615,786	99.06	7.59	99.13%
AP08	85,750,702	84,991,686	99.11	14.76	99.52%
AP09	78,778,208	77,967,505	98.97	13.56	99.49%
AP10	80,450,254	79,801,244	99.19	13.61	99.48%
FCG01	84,282,552	83,620,646	99.21	14.34	99.52%
FCG02	81,263,586	76,406,913	94.02	12.43	98.37%
FCG03	61,744,694	59,723,425	96.73	8.56	99.11%
FCG04	82,443,344	73,825,046	89.55	12.25	99.44%
FCG05	112,695,646	103,290,253	91.65	6.76	99.13%
FCG06	82,035,818	58,450,652	71.25	8.77	99.18%
FCG07	102,132,854	98,320,756	96.27	15.67	99.49%
FCG08	119,862,310	115,463,531	96.33	18.92	99.55%
FCG09	86,193,752	75,039,441	87.06	11.95	99.42%
FCG10	89,881,584	89,313,299	99.37	15.54	99.48%

**Table 4:** Population genetic statistics.

Sample	SNP Number	Transition	Transversion	Ti/Tv	Heterozygosity	Homozygosity	Het. Ratio
PT01	3,230,608	2,049,891	1,180,717	1.74	2,390,214	840,394	73.99
PT02	3,177,195	2,016,506	1,160,689	1.74	2,326,580	850,615	73.23
PT03	3,207,060	2,033,107	1,173,953	1.73	2,373,126	833,934	74
PT04	3,113,547	1,974,129	1,139,418	1.73	2,378,681	734,866	76.4
PT05	3,151,947	1,999,431	1,152,516	1.73	2,331,673	820,274	73.98
PT06	3,133,597	1,989,456	1,144,141	1.74	2,275,313	858,284	72.61
PT07	3,231,087	2,048,365	1,182,722	1.73	2,417,106	813,981	74.81
PT08	3,202,291	2,030,806	1,171,485	1.73	2,348,822	853,469	73.35
PT09	3,196,353	2,028,181	1,168,172	1.74	2,362,823	833,530	73.92
PT10	3,209,236	2,035,816	1,173,420	1.74	2,367,876	841,360	73.78
YJ01	3,204,803	2,035,452	1,169,351	1.74	2,346,537	858,266	73.22
YJ02	3,076,371	1,953,685	1,122,686	1.74	2,206,752	869,619	71.73
YJ03	2,949,881	1,872,735	1,077,146	1.74	2,071,177	878,704	70.21
YJ04	3,210,281	2,036,777	1,173,504	1.74	2,350,626	859,655	73.22
YJ05	3,190,676	2,025,521	1,165,155	1.74	2,332,962	857,714	73.12
YJ06	3,210,827	2,037,811	1,173,016	1.74	2,352,293	858,534	73.26
YJ07	3,183,752	2,018,315	1,165,437	1.73	2,326,536	857,216	73.08
YJ08	3,289,639	2,083,723	1,205,916	1.73	2,430,629	859,010	73.89
YJ09	3,145,002	1,997,742	1,147,260	1.74	2,281,389	863,613	72.54
YJ10	3,292,833	2,086,024	1,206,809	1.73	2,431,034	861,799	73.83
AP01	3,104,373	1,970,483	1,133,890	1.74	2,242,025	862,348	72.22
AP02	3,157,093	2,003,547	1,153,546	1.74	2,308,917	848,176	73.13
AP03	3,031,091	1,925,332	1,105,759	1.74	2,162,685	868,406	71.35
AP04	3,188,583	2,022,946	1,165,637	1.74	2,336,169	852,414	73.27
AP05	3,051,078	1,931,149	1,119,929	1.72	2,188,455	862,623	71.73
AP06	3,119,827	1,981,112	1,138,715	1.74	2,273,222	846,605	72.86
AP07	2,818,190	1,790,312	1,027,878	1.74	1,925,968	892,222	68.34
AP08	3,209,983	2,036,386	1,173,597	1.74	2,367,467	842,516	73.75
AP09	3,184,590	2,020,756	1,163,834	1.74	2,325,943	858,647	73.04
AP10	3,182,885	2,019,852	1,163,033	1.74	2,338,087	844,798	73.46
FCG01	3,200,608	2,030,877	1,169,731	1.74	2,343,023	857,585	73.21
FCG02	10,363,458	6,368,531	3,994,927	1.59	6,760,257	3,603,201	65.23
FCG03	2,835,017	1,799,018	1,035,999	1.74	1,946,792	888,225	68.67
FCG04	3,103,322	1,970,222	1,133,100	1.74	2,236,762	866,560	72.08
FCG05	2,695,031	1,712,297	982,734	1.74	1,953,310	741,721	72.48
FCG06	2,901,476	1,836,705	1,064,771	1.72	2,020,702	880,774	69.64
FCG07	3,207,612	2,036,591	1,171,021	1.74	2,351,474	856,138	73.31
FCG08	3,240,686	2,055,097	1,185,589	1.73	2,383,249	857,437	73.54
FCG09	3,108,411	1,972,768	1,135,643	1.74	2,243,918	864,493	72.19
FCG10	3,194,781	2,026,672	1,168,109	1.74	2,334,079	860,702	73.06

## Data Availability

Genome assemblies and raw sequence data from SRA were deposited in NCBI’s Assembly database under BioProject accession number PRJNA1175469. https://www.ncbi.nlm.nih.gov/bioproject/PRJNA1175469 (accessed on 21 October 2024).
